# Antimicrobial Resistance Patterns: Review of the Antibiogram of a Surgical Unit in a Public Tertiary Care Hospital of Pakistan

**DOI:** 10.7759/cureus.11159

**Published:** 2020-10-25

**Authors:** Usama Muhammad Kathia, Talha Munir, Fatima Fateh, Adeel Ahmad, Awais Amjad, Muhammad Farooq Afzal

**Affiliations:** 1 Department of Urology, Lahore General Hospital, Lahore, PAK; 2 Department of Medicine, Doctors Hospital, Lahore, PAK; 3 Department of Medicine, Tehsil Head Quarter Hospital, Phalia, PAK; 4 Department of Surgery, Lahore General Hospital, Lahore, PAK

**Keywords:** antibiogram, antimicrobial resistance, surgery

## Abstract

Introduction

Antimicrobial resistance (AMR) has become a challenge in modern-day medical practice. The pace at which microbes are becoming resistant to antibiotics is greater than the discovery of novel antimicrobial agents. There is a need to study these antimicrobial patterns and, for this purpose, antibiograms should be developed at the levels of wards and hospitals and studied to guide us better on how to choose suitable empirical therapy for our patients.

Methods and materials

A total of 286 reports were studied, which contained the culture and sensitivity data of all the patients admitted under the care of Surgical Unit-1 in Lahore General Hospital between April 1, 2019, and October 31, 2019. All the samples for culture and sensitivity were sent to the in-house laboratory of the hospital where the reporting was done by the pathology department. They were inoculated and then intubated, and gram staining was performed. Antibiotic resistance and susceptibility were measured by the disk diffusion method according to the Clinical and Laboratory standards institute (CSLI) guidelines.

Results

The most common isolated organism was *Escherichia coli​​​​​​*​ (*E. coli) *in 65 (24%) patients, the next most common was *Acinetobacter* *species* in 62 (23%), followed by *Pseudomonas species* 52(19%), *Klebsiella species* 32 (13%), *Staphylococcus aureus *30 (11%), *Coagulase-negative Staphylococci *20 (7%), *Enterobacter species* (2%), and *Citrobacter species* (1%). The antimicrobial susceptibility of *E. coli *was highest for aminoglycosides and carbapenems like amikacin (78%), meropenem (71%), and imipenem (63%). *Acinetobacter *was most sensitive to colistin (100%), amikacin (31%), meropenem (21%), and cefoperazone + sulbactam (21%). *Pseudomonas *was also most sensitive to colistin (93%) and after that amikacin (52%), meropenem (52%), and imipenem (44%). *Klebsiella* was most sensitive to colistin (86%), imipenem (60%), and aminoglycosides (50%). Among gram-positive organisms, *Staphylococcus aureus *was sensitive to linezolid (100%) and vancomycin (100%).

Conclusion

The vast majority of isolated organisms in this study were gram-negative bacteria, and most were showing high antimicrobial resistance. The antibiograms should be developed and regularly updated at every ward and hospital. There is a need to bring more awareness about the proper use of antimicrobials among healthcare workers, and antimicrobial stewardship programs can help in this matter.

## Introduction

Antimicrobial resistance (AMR) has become a challenge in modern-day medical practice. The pace at which microbes are becoming resistant to antibiotics is greater than the discovery of novel antimicrobial agents. This problem is substantially greater in developing countries like Pakistan with limited resources and public awareness [1]. Hence, there is a need to study these patterns. The way to do so is to develop antibiograms at the institutional or even ward level. Antibiograms function to guide the use of antibiotics for prophylactic and empirical purposes. They display the current trends of microbes isolated from different patients and the antibiotic resistance patterns of these isolates [2].

It is essential to develop and analyze these antibiograms so that physicians are better aware of the current trends of antimicrobial resistance in their respective wards and institutions. It also guides the development of antimicrobial stewardship programs, which may help tackle the problem of antimicrobial resistance in an organized manner [3].

The surgery departments cater to patients with a multitude of problems, where wounds are often involved. Thus, wound site infections are present in a major bulk of surgical patients. The use of antibiotics are of special importance in such a department, hence the need to develop and study an antibiogram of a surgical unit.

## Materials and methods

The study was approved by the ethical review committee of the Post Graduate Medical Institute in Lahore General Hospital, Lahore, Pakistan, with approval number 00-170-20. A total sample of 286 culture and sensitivity reports was taken from the patients of Surgical Unit-1 of Lahore General Hospital. The data were collected in a prospective manner. The sample contains all the culture and sensitivity reports sent from this surgical unit from April 1, 2019, to October 31, 2019. The patients under the care of this unit were admitted to wards, surgical high dependency units (HDUs), and surgical intensive care units (ICUs). All the samples were sent to the in-house laboratory in Lahore General Hospital where culture and sensitivity reporting was done in the microbiology section of the pathology department. Samples used in the process were tracheal secretions, wound tissue, urine, blood, central venous pressure (CVP) tip, sputum, cerebrospinal fluid (CSF), and fluids. All samples were inoculated and then intubated, and gram staining was performed. Antibiotic resistance and susceptibility were measured by the disk diffusion method according to the Clinical and Laboratory Standards Institute (CSLI) guidelines. The reports were obtained from the central hospital information system.

## Results

**Figure 1:**
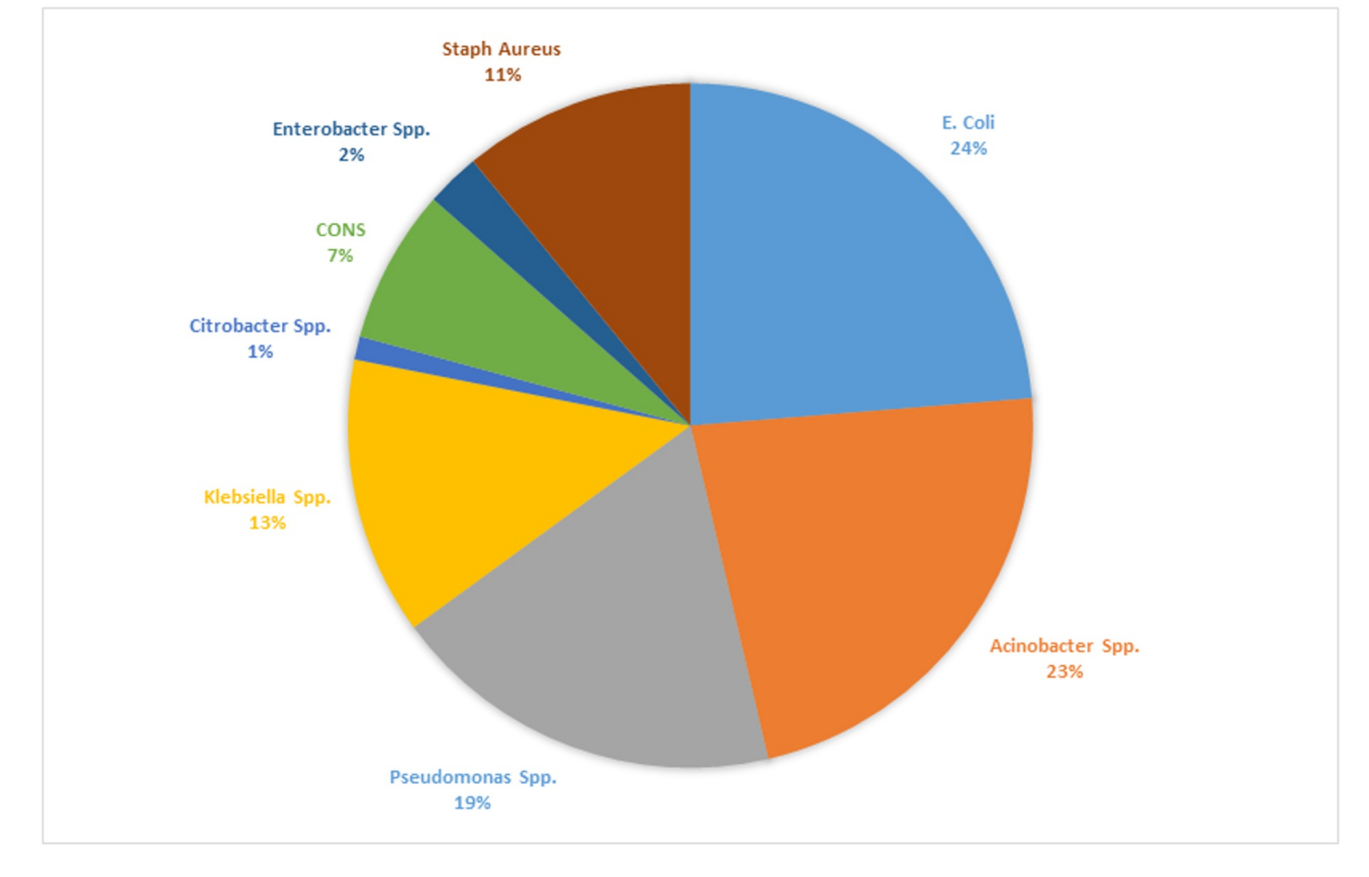
Percentages of all the microbes isolated from Surgical Unit-1 of Lahore General Hospital

Among the microorganisms, the most common isolate was* Escherichia coli* (*E.coli)*,* *which was present in 65 (24%) patients. The next most common organism was *Acinetobacter species *in 62 (23%) patients. The other most commonly isolated organisms were also gram-negative, which included Pseudomonas species (19%) and *Klebsiella species *(13%). Gram-positive bacteria were present in fewer patients, and the most common among them were *Staphylococcus aureus *and coagulase-negative *Staphylococci, *with 30 (11%) and 20 (7%) reports respectively. Other isolated organisms were *Enterobacter species *(2%) and *Citrobacter species *(1%) (Figure 1).

**Figure 2:**
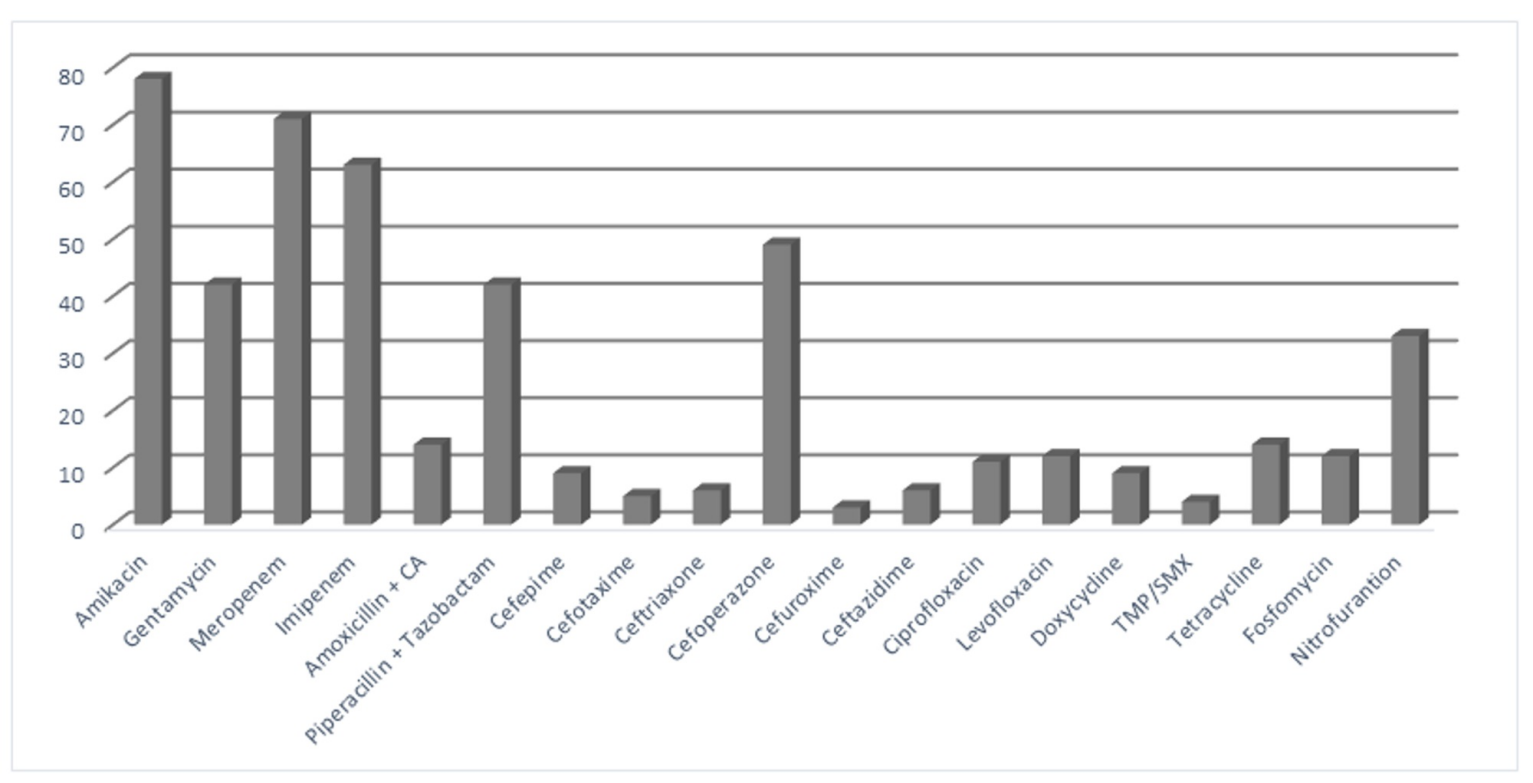
Antibiotic susceptibility pattern of E. coli

The antimicrobial susceptibility of *E. coli* was highest for carbapenems and aminoglycosides like amikacin (78%), meropenem (71%), and imipenem (63%) (Figure 2).

**Figure 3:**
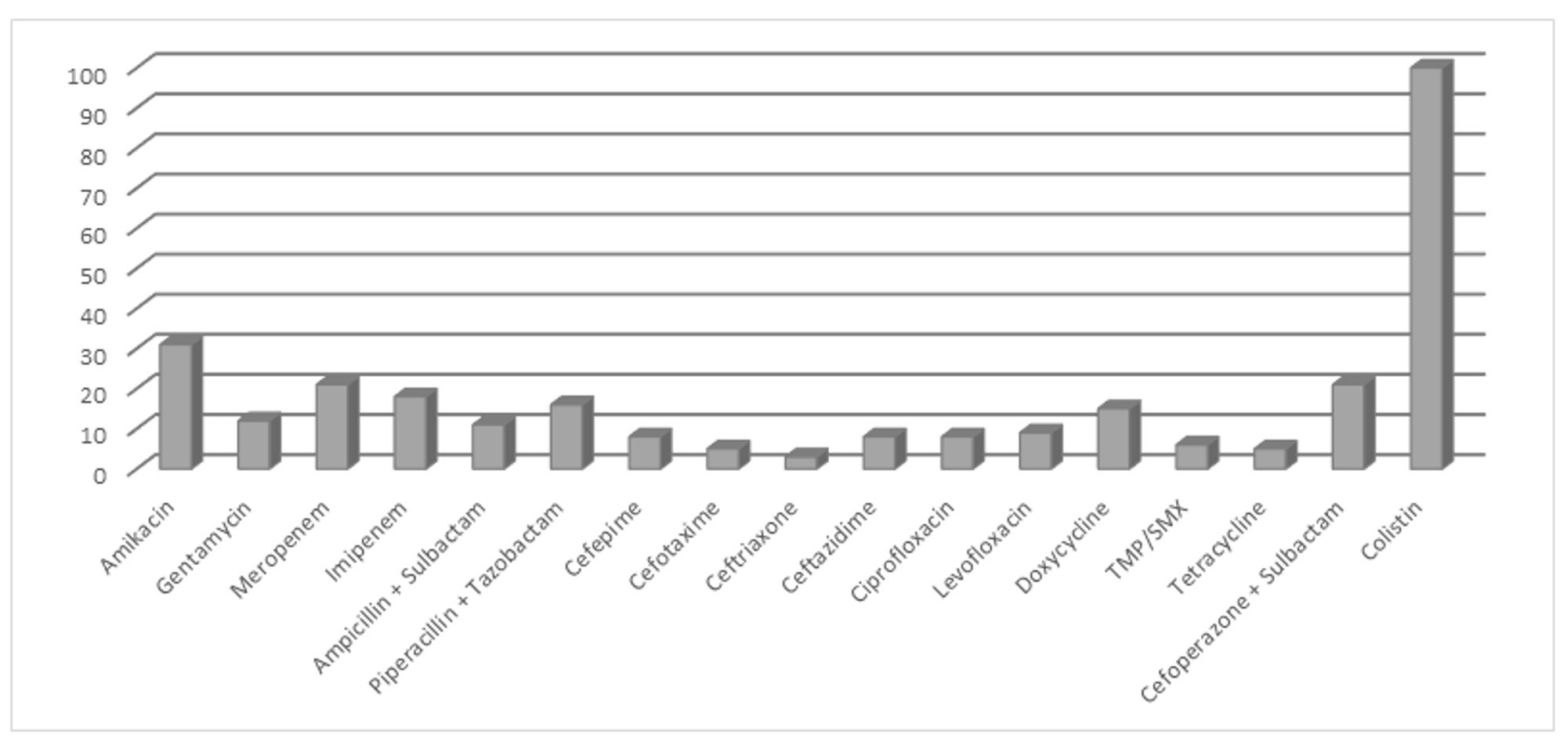
Antibiotic susceptibility pattern of Acinetobacter

Acinetobacter species was most sensitive to colistin (100%), aminoglycosides like amikacin (31%), meropenem (21%), and cefoperazone + sulbactam (21%) (Figure 3).

**Figure 4:**
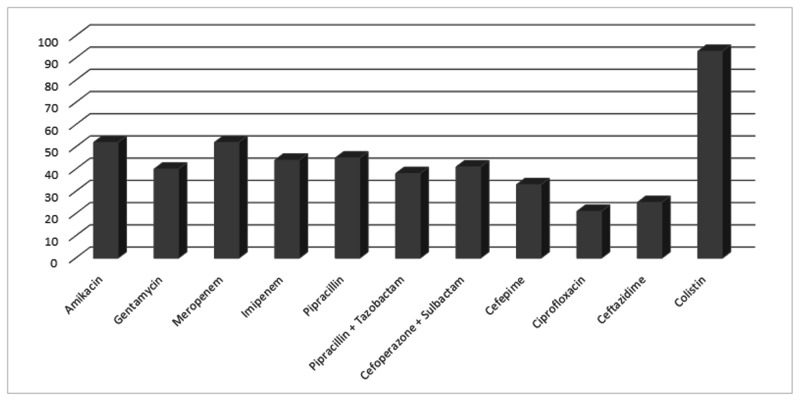
Antibiotic susceptibility pattern of Pseudomonas

*Pseudomonas species *was also most sensitive to colistin (93%) and after that aminoglycosides and carbapenems like amikacin (52%), meropenem (52%), and imipenem (44%) (Figure 4).

**Figure 5:**
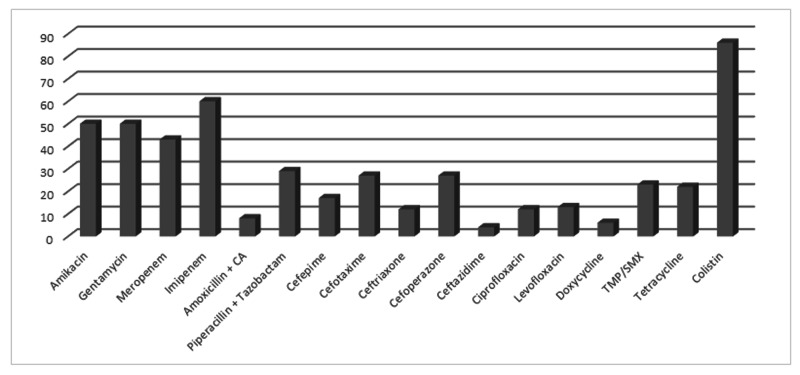
Antibiotic susceptibility pattern of Klebsiella

*Klebsiella species *was most sensitive to colistin (86%). Sensitivity to imipenem and aminoglycosides was 60% and 50%, respectively (Figure 5).

**Figure 6:**
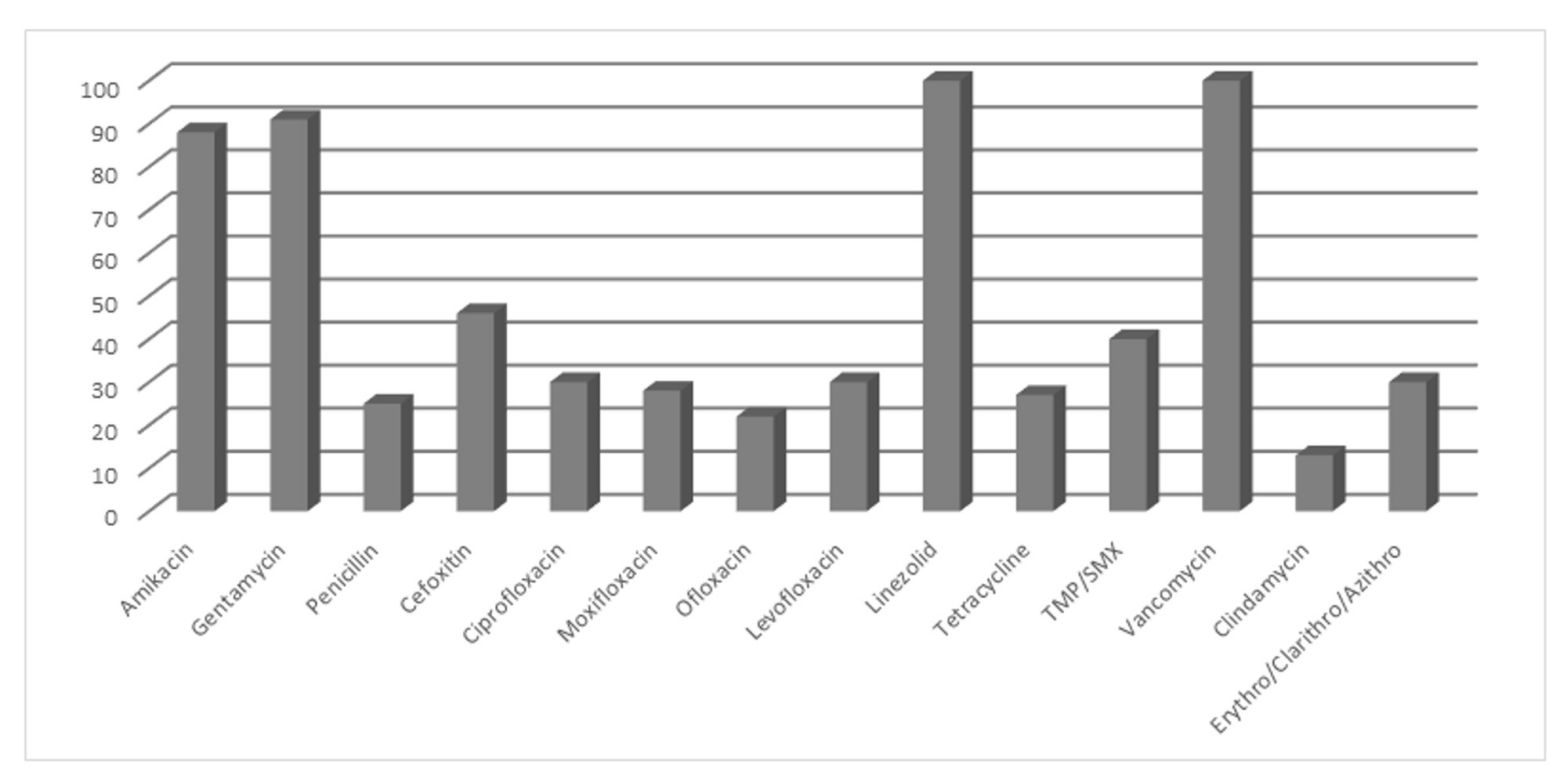
Antibiotic susceptibility pattern of Staphylococcus aureus

Among gram-positive organisms, *Staphylococcus aureus *was sensitive to linezolid (100%) and vancomycin (100%). It also showed high susceptibility toward aminoglycosides (Figure 6).

**Figure 7:**
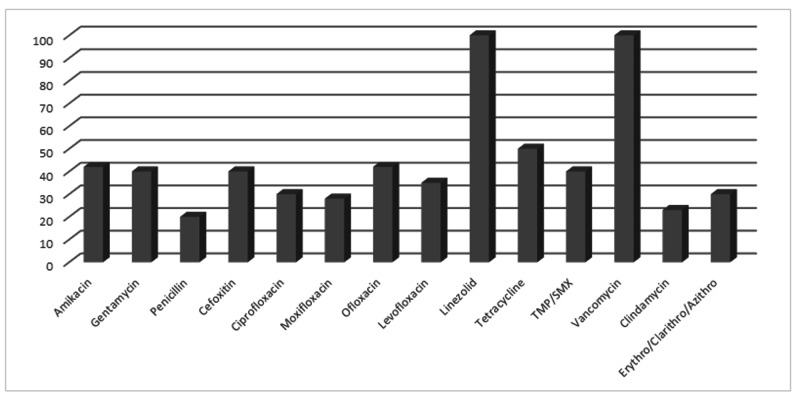
Antibiotic susceptibility pattern of CONS CONS: coagulase-negative Staphyloccocus species

Coagulase-negative* Staphyloccocus species *was also highly sensitive to linezolid (100%) and vancomycin (100%) but had a poorer response to aminoglycosides (41%) (Figure 7).

Different organisms were isolated from different specimens. The most commonly sent sample was a wound sample in which the most commonly isolated organism was *E.coli *(53) followed by *Acinetobacter species *(43) and *Pseudomonas species *(41). The most common isolate in tracheal secretions was *Acinetobacter species *(9). In urine samples, *Acinetobacter species *and *E.coli *were both isolated three times (Table 1).

**Table 1:** Distribution of isolated organisms from different specimens ETT: endotracheal tube; CSF: cerebrospinal fluid; CVP: central venous pressure; MRSA: methicillin-resistant Staphylococcus aureus

Bacteria	Specimen	Wound	ETT	CSF	Fluid	Blood	CVP	Sputum	Urine
Pseudomonas species	52	41	6	1	2	0	0	0	2
Citrobacter species	3	2	1	0	0	0	0	0	0
Enterobacter species	7	6	0	0	1	0	0	0	0
Enterococcus species	1	0	0	0	0	0	0	0	1
E. coli	65	53	0	2	7	0	0	0	3
Providencia species	1	1	0	0	0	0	0	0	0
Proteus mirabilis	1	1	0	0	0	0	0	0	0
Klebsiella species	32	20	5	0	3	2	0	0	2
Klebsiella pneumoniae	3	2	1	0	0	0	0	0	0
Klebsiella oxytoca	1	1	0	0	0	0	0	0	0
Morganella morganii	1	0	0	0	1	0	0	0	0
Acinetobacter species	62	43	9	0	1	2	1	3	3
Staphylococcus species	14	10	0	0	0	3	1	0	0
MRSA	9	8	0	0	0	1	0	0	0
Staphylococcus aureus	24	21	1	0	1	1	0	0	0
Proteus species	10	7	3	0	0	0	0	0	0

## Discussion

The choice of empirical antimicrobial therapy is made on the basis of local guidelines and antibiogram patterns that demonstrate the trends of antimicrobial resistance and organisms commonly isolated from a particular hospital or ward. Hence, it is very important for the physician to familiarize himself with this information. With time, newer trends of antimicrobial resistance are being discovered, so the patterns of antibiotic resistance are dynamic in nature [4]. That’s the reason antibiograms are regularly updated based upon hospital protocols.

In our study, a majority of isolates were gram-negative bacteria. The most common one was *E. coli*, making up almost a quarter of our total sample with a presence in 65 specimens. A study by Esposito et al. also observed *E. coli *to be the most prevalent organism [5]. It was most commonly found in wounds. *E. coli *was highly sensitive to aminoglycosides and carbapenems with sensitivity towards amikacin at 78% and meropenem at 71%. In a similar study by Rajan et al., high sensitivity was observed toward aminoglycosides and carbapenems with sensitivity towards amikacin at 82% and imipenem at 93% [6]. A study by Qadeer et al. also showed 93% sensitivity toward amikacin and 90% towards carbapenems (meropenem and imipenem). But the most sensitivity in their study was shown toward colistin (100%) [7]. Ahmad et al. also demonstrated high sensitivity towards amikacin (80%) and imipenem (100%) [8].

The second most common bacteria isolated in this study is *Acinetobacter species*. It showed 100% sensitivity toward colistin, but it was highly resistant toward all other drugs with sensitivity toward meropenem and cefoperazone + sulbactam at 21% and aminoglycosides like amikacin at 31%. This high resistance toward carbapenems was also observed by Qadeer et al., who showed 100% resistance toward them. They also demonstrated a 5% sensitivity towards amikacin. Similarly, Rajan et al. also showed a 48% sensitivity toward carbapenems. Gill et al. showed a 41% sensitivity of *Acinetobacter *towards doxycycline and a 100% resistance toward meropenem and third-generation cephalosporins [9]. Esposito et al., in their study, also registered high resistance toward carbapenems [5]. Similar to our study, the most sensitivity is shown towards colistin by Qadeer and Rajan [6-7], whereas Ahmad and Esposito showed the highest sensitivity of tigecycline towards *Acinetobacter*. There has been a lot of concerns regarding the multidrug resistance toward *Acinetobacter* and its role in nosocomial infections. A study by Hasan et al. has demonstrated the high prevalence of *Acinetobacter Baumani* in different hospitals of Pakistan [10]. Their results concurred with our finding of high carbapenem resistance in this bacteria. According to Hasan et al., the drug most sensitive to *Acinetobacter* is tigecycline (80%) followed by Colistin (50%). In our study, *Acinetobacter* was found in the majority of tracheal secretions similar to the study by Qadeer and Ahmad [7-8].

The third most common bacteria isolated was *Pseudomonas species*. It was most sensitive toward colistin at 93% followed by meropenem and amikacin at 52% and imipenem at 44%. The majority was seen in wounds followed by tracheal secretions. It was also the third most common isolate in the study by Gill et al., who showed a 64% sensitivity toward meropenem and 50% toward amikacin. The most sensitivity in their study was shown by polymyxin-B at 100% followed by piperacillin + tazobactam at 71% [9]. Our study showed only 38% sensitivity towards piperacillin + tazobactam. According to Rajan et al., carbapenems' sensitivity to *Pseudomonas* was 87% and amikacins' 61% [6]. Similar to our study, the highest sensitivity toward colistin was shown by Qadeer et al. [7].

The next most common bacteria is *Klebsiella species*. It was the third most common bacteria in tracheal secretions. It was most sensitive to colistin 86%, imipenem 60%, and aminoglycosides 50%. It was the most common bacteria reported by Rajan et al; he demonstrated 71.87% sensitivity toward carbapenem [6]. Esposito et al. demonstrated high sensitivity towards carbapenem [5]. Sheth et al. demonstrated 100% sensitivity towards carbapenem. Our study also showed high resistance toward third-generation cephalosporins.

The most common gram-positive organism in this study was *Staphylococcus aureus, *which was the fifth most common microbe overall. A vast majority of this microbe was isolated from the wound tissue specimen. It showed a 100% sensitivity toward linezolid and vancomycin. It was also highly sensitive towards aminoglycosides like amikacin (88%) and gentamycin (91%). The study by Esposito et al. demonstrated high sensitivity toward oxacillin, whereas we found high resistance toward penicillins. Out of a total of 24 isolates, nine (37.5%) were designated as methicillin-resistant Staphylococcus​​ aureus​​​​​ (MRSA). MRSA is a nuisance in any hospital and has the ability to prolong hospital stay and the cost of treatment for the patient. Hence, its prevention and treatment should be of utmost priority for any healthcare facility [11].

These data demonstrate an example of a huge problem in the healthcare of low to middle-income countries that is antimicrobial resistance (AMR). The misuse of antibiotics has been thought of as the main factor behind this problem with a lack of strict regulations on the buying and selling of antibiotics to a lack of awareness among healthcare workers of their cautious and proper use. A study by Alavi et al. showed that 45% of the prescribed antibiotics were not required, and 13% were given an inappropriate dosage [12]. It is important to bring more awareness towards AMR among the healthcare staff and one useful way of doing it is by starting antimicrobial stewardship programs in hospitals. These programs have demonstrated a reduction in the cost of treatment and hospital stay for surgical patients, with no effect on mortality [13]. They have also demonstrated a reduction in the use of antibiotics in critical care patients without any effect on mortality [14].

## Conclusions

AMR is a growing problem in low to middle-income countries. It is important to understand the local prevalence of most common organisms isolated from different specimens and their antibiotic susceptibilities. For this purpose, it is important to develop antibiograms at the levels of hospitals and wards. It can help the physicians to choose empirical antimicrobials according to the local trends of AMR. In the study of antibiograms of Surgical Unit-1 of Lahore General Hospital, it was found that the majority of the isolates are gram-negative bacteria, with *E. coli *and *Acinetobacter species *making up the majority. Also, there is a need to develop antimicrobial stewardship programs in hospitals to tackle the misuse of antimicrobials.
